# Characterization of novel arrhythmogenic patterns arising secondary to heterogeneous expression and activation of Nav1.8

**DOI:** 10.3389/fcvm.2025.1546803

**Published:** 2025-03-20

**Authors:** Zhong-He Zhang, Hector Barajas-Martinez, Hong-Yi Duan, Guo-Hua Fan, Hong Jiang, Charles Antzelevitch, Hao Xia, Dan Hu

**Affiliations:** ^1^ Department of Cardiology, Renmin Hospital of Wuhan University, Wuhan, China; ^2^ Cardiovascular Research Institute, Wuhan University, Wuhan, China; ^3^ Hubei Key Laboratory of Cardiology, Wuhan, China; ^4^ Cardiovascular Research Department, Lankenau Institute for Medical Research, Lankenau Heart Institute, Wynnwood, PA, United States; ^5^ Sidney Kimmel Medical College, Thomas Jefferson University, Philadelphia, PA, United States; ^6^ Department of Thoracic Surgery, Renmin Hospital of Wuhan University, Wuhan, China

**Keywords:** *SCN10A*, pharmacology, arrhythmia, Purkinje fibers, cardiac electrophysiology

## Abstract

**Background:**

Previous studies suggested that *SCN10A*/Nav1.8 may influence cardiac electrophysiology and the susceptibility to cardiac arrhythmias. Notably, the expression of *SCN10A* is not uniform, showing variable expression in each cardiac chamber. The present study aims to explore the functional significance of Nav1.8 expression among different cell types present in the ventricular myocardium.

**Methods:**

The effect of the specific Nav1.8 blocker, A-803467, on action potential was recorded from epicardial, mid-myocardial (M cells) and Purkinje tissue slices isolated from the canine left ventricle using standard microelectrode techniques and on late sodium current from Purkinje cells using patch-clamp techniques.

**Results:**

A-803467 treatment did not significantly affect maximum diastolic potential, action potential amplitude or maximum rate of rise of the action potential upstroke in epicardial cells, M cells or Purkinje fibers. Action potential duration (APD) was also unaffected by A-803467 in epicardial cells. However, administration of 1,000 nmol/L A-803467 reduced APD_30_, APD_50_, and APD_90_ during relatively slow pacing rates of 0.2 and 0.5 Hz in M cells. In Purkinje fibers, A-803467 (100 and 1,000 nmol/L) substantially abbreviated APD_50_ and APD_90_ at slow pacing rates (0.2 and 0.5 Hz). Moreover, 100 nmol/L A-803467 significantly inhibited the development of early afterdepolarizations induced by 10 nmol/L ATX-II (7/8 vs. 2/8, *p* < 0.05) as well as the amplitude of late sodium current at 0.2 Hz in Purkinje cells.

**Conclusions:**

The functional significance of Nav1.8 varies among different types of ventricular and conduction system cardiomyocytes. The reduction in I_Na,L_ and APD, as well as suppression of early afterdepolarizations by Nav1.8 block in Purkinje fibers suggests Nav1.8 as a potential therapeutic target for bradycardia-dependent arrhythmias.

## Introduction

The voltage-gated sodium channel 1.5 (Nav1.5), encoded by *SCN5A* gene is recognized as the primary cardiac Nav isoform. Mutations in Nav1.5 have been associated with a variety of cardiac disorders, including long QT syndrome (LQTS), Brugada syndrome, cardiac conduction system disease and atrial fibrillation (AF) (1, 2). However, other sodium channels including Nav1.8 are also present in the heart. Nav1.8 is a tetrodotoxin-resistant voltage-gated sodium channel, initially identified as predominantly expressed in small-diameter sensory neurons of the dorsal root ganglia, where it plays a crucial role in pain perception and modulation (3).

Several genome-wide association studies have demonstrated that common single nucleotide polymorphisms and variants in *SCN10A* are associated with electrocardiographic indices, including PR, QRS and QT interval duration, as well as cardiac arrhythmia phenotypes, such as Brugada syndrome, AF, cardiac conduction system disease and sudden unexpected death syndrome (4–10). The expression of *SCN10A* mRNA and Nav1.8 protein has been detected in atrial and ventricular myocytes of mice, rabbits, and humans (4, 11–16). Moreover, pharmacological block of Na1.8 channels using the highly selective inhibitor A-803467 has been shown to prolong both PR interval and QRS duration in wild-type mice (4). Moreover, Yang et al. reported that application of A-803467 in ventricular cardiomyocytes of mice and rabbits results in abbreviated action potential duration (APD) as well as suppression of early afterdepolarizations (EADs) (15). These findings collectively suggest that *SCN10A* plays an important role in cardiac electrophysiology and arrhythmogenesis.

The distribution of *SCN10A* exhibits heterogeneity among atrial and ventricular tissues, as well as between the left and right ventricles. Yang et al. also reproted that Nav1.8-mediated late sodium currents (I_Na,L_) varied among individual ventricular cardiomyocytes in mice. Furthermore, the APD-shortening effect of A-803467 demonstrated considerable cell-to-cell variability, with only approximately half of the tested cells showing significant reduction in APD (15). Accordingly, the physiological effects mediated by Nav1.8 may differ among the different cell types. The canine ventricular wall encompasses epicardial cells, M cells, and Purkinje cells, each exhibiting distinct electrophysiological and pharmacological properties. However, the electrophysiological characteristics of Nav1.8 across these cell subtypes remain unexplored. This study aims to elucidate the functional electrophysiological role of Nav1.8 in different cellular subtypes of the ventricular myocardium.

## Materials and methods

### Slice preparation and Purkinje fibers

All experiments involving animals were approved by the Animal Care and Use Committee of the Renmin Hospital of Wuhan University. All animal procedures conformed to the Guidelines for the Care and Use of Laboratory Animals outlined by the US National Institutes of Health. Adult mongrel dogs weighing 20–35 kg were anticoagulated with heparin and anesthetized with sodium pentobarbital (30 mg/kg, intravenous). The chest was opened via a left thoracotomy, and the heart was rapidly excised and placed in a cardioplegic solution (4℃ Tyrode solution containing 12 mmol/L KCl).

Epicardial (<1.5 mm from epicardial surface) and mid-myocardial (2–7 mm from epicardial surface) tissues were isolated from the left ventricle of the hearts by razor blade shavings (Davol Simon Dermatome, Cranston, RI, USA) made parallel to the surface of the ventricular free wall. Free running Purkinje fibers were dissected out from left ventricle. The preparations were placed in a tissue bath (volume 5 ml, flow rate 12 ml/min) and allowed to equilibrate for at least 3 h while superfused with Tyrode's solution bubbled with 95% O_2_-5% CO_2_ and maintained at (37 ± 0.5℃, pH = 7.35). The composition of the Tyrode's solution was (in mmol/L): NaCl 129, KCl 4, NaH_2_PO_4_ 0.9, NaHCO_3_ 20, CaCl_2_ 1.8, MgSO_4_ 0.5, and D-glucose 5.5.

### Isolation of Purkinje cells

Purkinje cells were isolated via enzymatic dissociation as previously described (17). Purkinje fibers were dissected from the left ventricles and placed in a small dish. Purkinje fibers were then digested in a nominally Ca^2+^-free solution containing 1.0 mg/ml collagenase (type II, Worthington) and 30 mM 2,3-butanedione monoxime at 36°C. Gentle stirring of the enzyme solution with a small magnetic bar facilitated the release of cells from the fibers. The suspension containing dissociated Purkinje cells was periodically collected and transferred into a modified KB solution, while fresh enzyme solution was added to the remaining Purkinje fibers to maintain a volume of 2 ml. The enzymatic digestion process typically took 15–35 min to yield individual myocytes. Finally, the isolated Purkinje cells were stored in the modified KB solution at room temperature until further use.

### *SCN10A* plasmid transfection in ND7/23 cells

The wild-type cDNA plasmid (2 µg) of *SCN10A*-encoded Nav1.8 channel and green fluorescent protein (0.5 µg) were co-transfected into ND7/23 cells with 10 µl FuGene6 in culture for 48 h. Then green fluorescent protein colored cells were selected as successful transfection for patching clamp experiments to verify the effect of the drug A-803467 (see below) on I_Na,L_.

### Action potential (AP) recordings

The preparations were stimulated at basic cycle lengths (BCLs) ranging from 500 to 5,000 ms. Standard techniques were used to deliver rectangular-wave pulses lasting 1–3 ms at 2.5 times diastolic threshold intensity through silver bipolar electrodes that were insulated except at the tips.

Transmembrane potentials were recorded using 3 mmol/L KCL-filled glass microelectrodes (tip resistances, 10–20 MΩ) connected to a high input-impedance amplification system (World Precision Instruments, New Haven, USA.) The signals were displayed on oscilloscopes and further amplified, digitized and analyzed (Spike 2, Cambridge Electronic Design, Cambridge, England). The following action potential (AP) parameters were evaluated before and after exposure to A-803467 (for 20–30 min): action potential amplitude (APA), maximum diastolic potential (MDP), and maximum rate of rise of the AP upstroke (Vmax), APD at 30%, 50% and 90% repolarization (APD_30_, APD_50_ and APD_90_). ATX-II (10 nmol/L, for 20–30 min) was used to induce EADs in canine Purkinje fibers. EADs, characterized by depolarizing oscillations during phase 2 or 3 of the action potential, were evaluated at BCLs ranging from 500 to 5,000 ms.

### I_Na,L_ recordings

I_Na,L_ was recorded using the whole-cell patch-clamp technique. The bath solution used contained (in mmol/L): NaCl 140, CaCl_2_ 2.0, MgCl_2_ 1, glucose 10, and HEPES 10, pH adjusted to 7.4 with NaOH. 10 μmol/L nifedipine was added in bath solution to block calcium current. The pipette solution was composed of (in mmol/L): NaCl 10, aspartate 130, MgCl_2_ 1, CsCl 10, HEPES 10, MgATP 5, and EGTA 10, pH adjusted to 7.2 with CsOH. I_Na,L_ was assessed by applying a 500-ms square depolarization pulse to −20 mV from a holding potential of −80 mV, repeated every 5 s. The amplitude of I_Na,L_ was measured at the termination of the testing depolarizing pulse. To record Nav1.8-mediated I_Na,L_ in ND7/23 cells, which inherently express tetrodotoxin-sensitive fast sodium currents, 30 nmol/L tetrodotoxin (for 20–30 min) was administered to selectively inhibit the endogenous currents.

### Drugs

A-803467 and ATX-II were purchased from Sigma-Aldrich Co (St. Louis, Missouri, USA). A-803467 was dissolved in 5% dimethyl sulfoxide, stored at a concentration of 50 mmol/L, and diluted to the desired final concentration prior to use. ATX-II was dissolved in distilled water to create a stock solution of 200 nmol/L, and then diluted to a final concentration of 10 nmol/L for use.

### Statistical analysis

All data are presented as mean ± standard error. The statistical analysis of the electrophysiological effects of A-803467 was conducted using one-way repeated measure analysis variance followed by Bonferroni's test or with a student *t* test. The incidence of EADs was assessed by using the Fisher exact test. The SPSS (version 26.0) and GraphPad Prism software (version 10.0) were used for data analysis and graphing. *P* < 0.05 was considered statistically signiﬁcant.

## Results

### Effect of A-803467 on AP characteristics in epicardial cells

**Figure 1:**
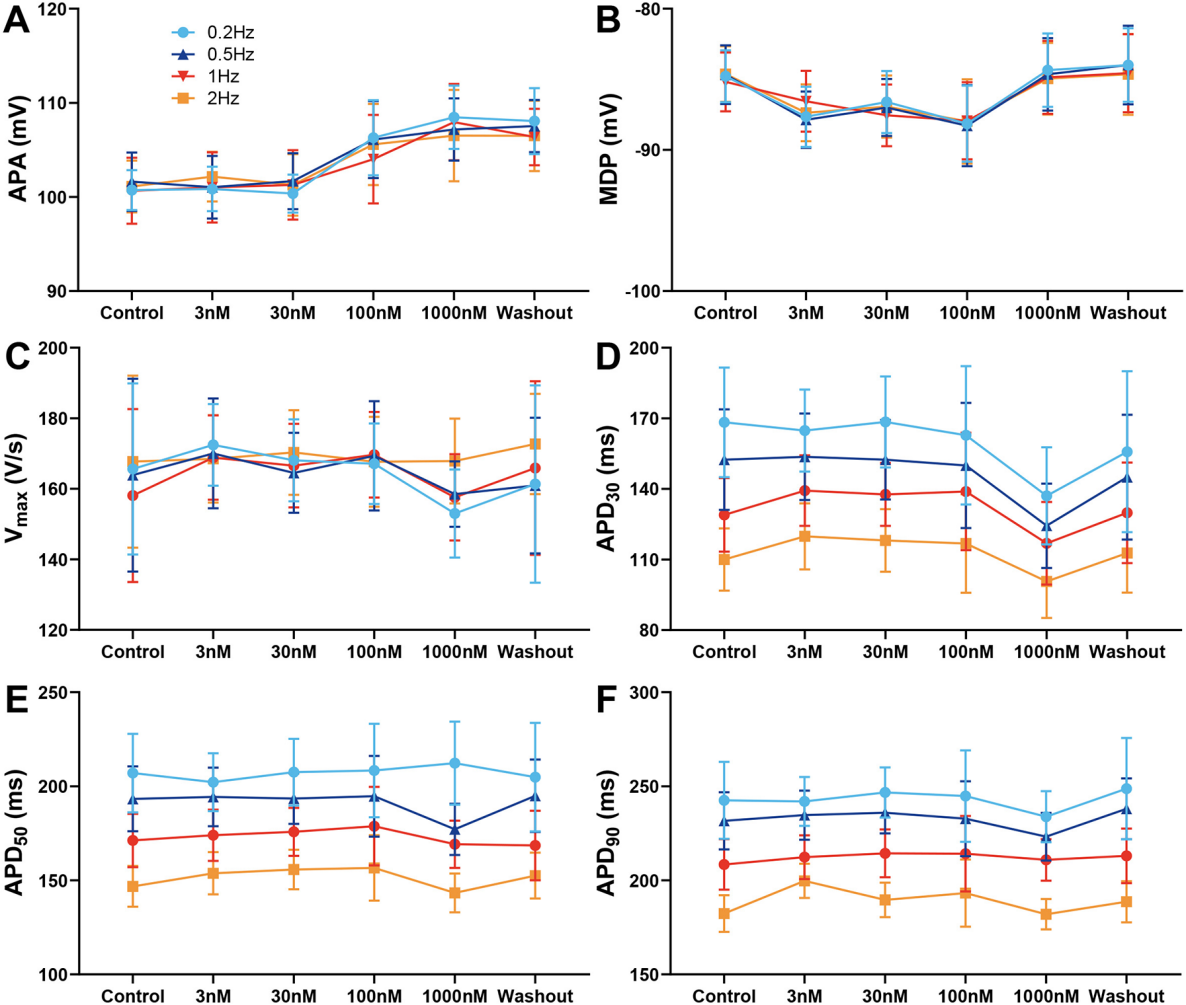
Effect of A-803467 (3–1,000 nmol/L) administration on action potential properties in canine left ventricular epicardial cardiomyocytes (*N* = number of dogs; *n* = number of tissue slices). **(A–F)** Average data for action potential amplitude (APA), maximum diastolic potential (MDP), maximum rate of rise of the action potential upstroke (Vmax), action potential duration at 30%, 50% and 90% repolarization (APD_30_, APD_50_ and APD_90_), before (control) and after administration and washout of A-803467 (*N* = 5 and *n* = 5, respectively).

We first assessed the effects of the Na_V_1.8 specific blocker A-803467 on AP characteristics in canine left ventricular epicardial preparations at BCLs from 500 to 5,000 ms. A-803467 (3–1,000 nmol/L) had no significant effect on APA, MDP, and Vmax (Figure 1A–C) at any BCL tested. Treatment with 1,000 nmol/L A-803467 exhibited a trend towards abbreviation of APD_30_ at stimulation BCLs ranging from 500 to 5,000 ms. However, this reduction was not statistically significant (Figure 1D). Both APD_50_ and APD_90_ showed no substantial change under the same conditions (Figure 1E,F).

### Effect of A-803467 on AP characteristics in M cells

**Figure 2:**
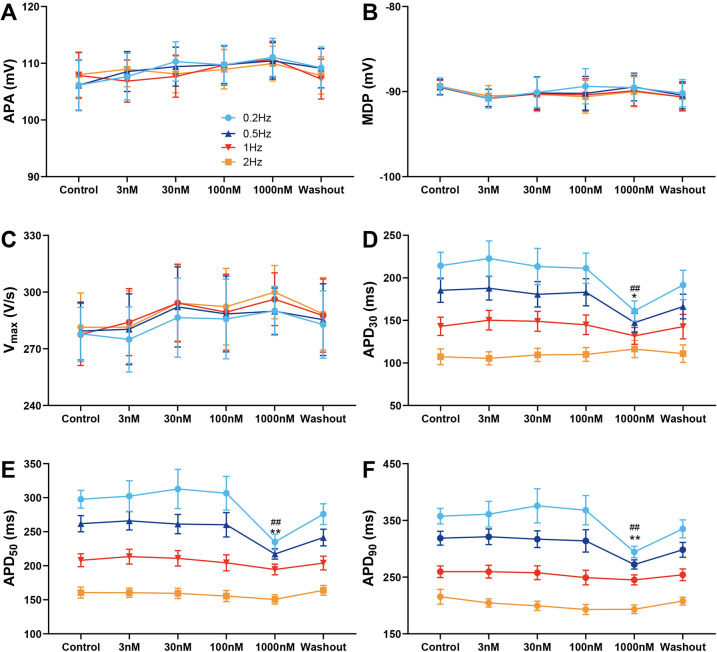
Effect of A-803467 (3–1,000 nmol/L) administration on action potential properties in canine left ventricular M cardiomyocytes (*N* = number of dogs; *n* = number of tissue slices). **(A–F)** Average data for action potential amplitude (APA), maximum diastolic potential (MDP), maximum rate of rise of the action potential upstroke (Vmax), action potential duration at 30%, 50% and 90% repolarization (APD_30_, APD_50_ and APD_90_), before (control) and after administration and washout of A-803467. *p < 0.05 vs. control at 0.5 Hz, **p < 0.01 vs. control at 0.5 Hz, ^#^*p* < 0.05 vs. control at 0.2 Hz, ^##^*p* < 0.01 vs. control at 0.2 Hz (*N* = 10 and *n* = 10, respectively).

Figure 2 illustrates average APs recordings at baseline, during exposure to increasing concentrations of A-803467, and following drug wash-out. Similarly to its effects on epicardial cells, APA, MDP, and Vmax were not altered by A-803467 exposure at concentrations ranging from 3 to 1,000 nmol/L at different BCLs (Figure 2A–C). At BCLs of 500 and 1,000 ms, A-803467, even at concentrations up to 1,000 nmol/L, did not produce significant changes in APD_30_, APD_50_, or APD_90_ compared with control group (Figure 2D–F). However, at BCLs of 2,000 and 5,000 ms, 1,000 nmol/L A-803467 significantly decreased APD_30_ (BCL = 2,000 ms: 185.39 ± 14.21 ms vs. 147.34 ± 10.99 ms, *p* < 0.05; BCL = 5,000 ms: 214.29 ± 15.81 ms vs. 161.09 ± 11.76 ms, *p* < 0.01), APD_50_ (BCL = 2,000 ms: 261.84 ± 11.87 ms vs. 217.33 ± 7.61 ms, *p* < 0.01; BCL = 5,000 ms: 297.72 ± 13.07 ms vs. 237.07 ± 9.03 ms, *p* < 0.01), and APD_90_ (BCL = 2,000 ms: 318.62 ± 12.21 ms vs. 272.23 ± 8.11 ms, *p* < 0.01; BCL = 5,000 ms: 357.51 ± 13.58 ms vs. 294.29 ± 10.09 ms, *p* < 0.01) (Figure 2D–F). These effects were reversed following wash-out of the drug. At BCLs of 2,000 and 5,000 ms, the abbreviating effects on APD were not observed when A-803467 was administered at concentrations from 3 to 100 nmol/L.

### Effect of A-803467 on AP characteristics, EADs and I_Na,L_ in Purkinje fibers

**Figure 3:**
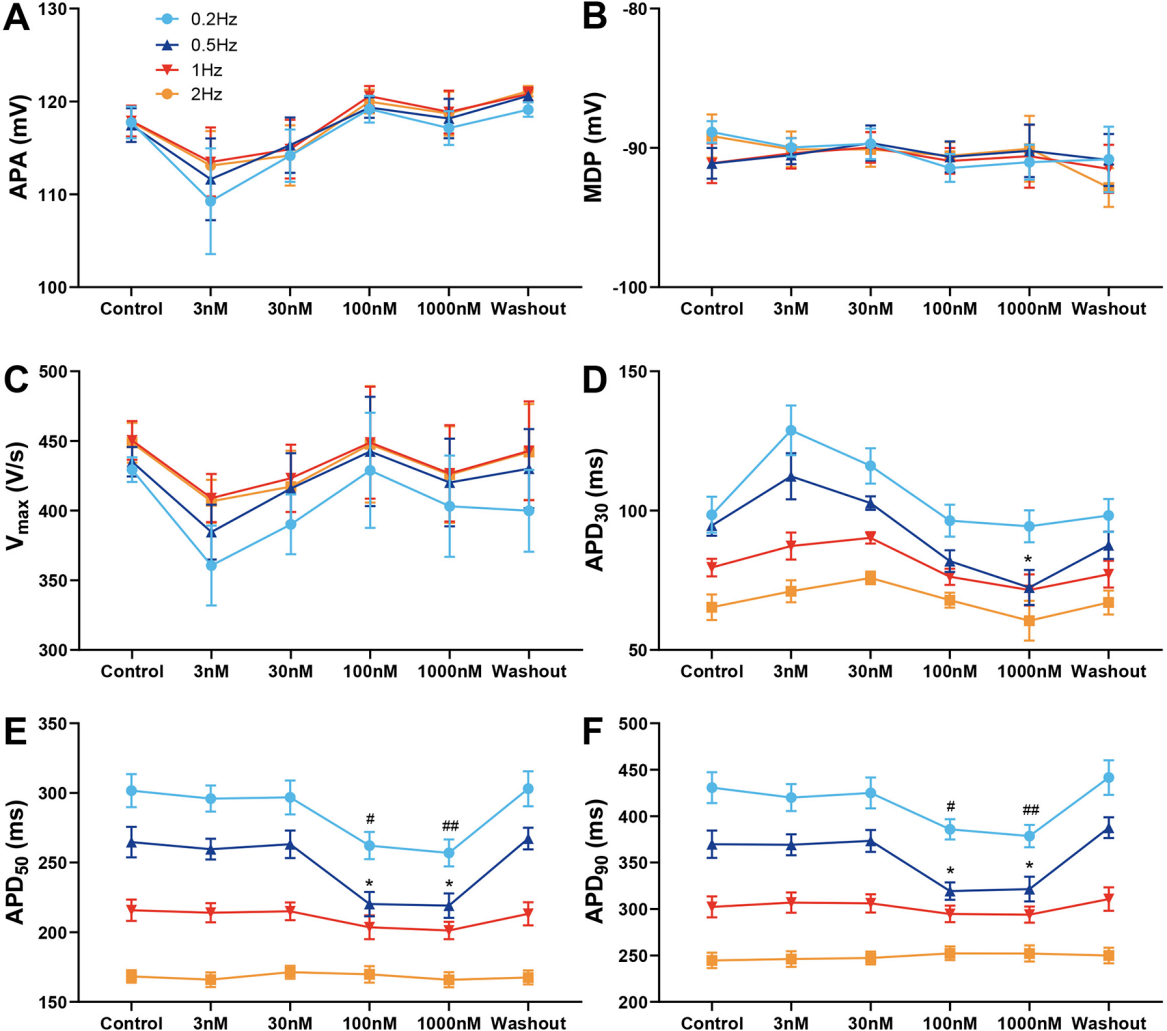
Effect of A-803467 (3–1,000 nmol/L) administration on action potential properties in canine left ventricular Purkinje fibers (*N* = number of dogs; *n* = number of tissue slices). **(A–F)** Average data for action potential amplitude (APA), maximum diastolic potential (MDP), maximum rate of rise of the action potential upstroke (Vmax), action potential duration at 30%, 50% and 90% repolarization (APD_30_, APD_50_ and APD_90_), before (control) and after administration and washout of A-803467. *p < 0.05 vs. control at 0.5 Hz, **p < 0.01 vs. control at 0.5 Hz, ^#^*p* < 0.05 vs. control at 0.2 Hz, ^##^*p* < 0.01 vs. control at 0.2 Hz (*N* = 6 and *n* = 6, respectively).

Figure 3 shows the effects of various concentrations of A-803467 on APs recorded in Purkinje fibers. No significant changes in APA, MDP and Vmax were observed at any BCL or concentration studied (Figure 3A–C). At BCLs of 500 and 1,000 ms, treatment with A-803467 did not significantly reduce APD_30_, APD_50_, or APD_90_ (Figure 3D–F). However, at a BCL of 2,000 ms, 100 nmol/L A-803467 remarkably reduced APD_50_ (264.73 ± 10.92 ms vs. 220.20 ± 8.69 ms, *p* < 0.05) and APD_90_ (369.73 ± 14.67 ms vs. 319.34 ± 9.43 ms, *p* < 0.05). Additionally, 1,000 nmol/L A-803467 significantly decreased APD_30_ (94.64 ± 3.63 ms vs. 72.42 ± 6.29 ms, *p* < 0.05), as well as APD_50_ (264.73 ± 10.92 ms vs. 219.14 ± 8.79 ms, *p* < 0.05) and APD_90_ (369.73 ± 14.67 ms vs. 321.51 ± 13.25 ms, *p* < 0.05). At a BCL of 5,000 ms, both 100 and 1,000 nmol/L A-803467 diminished APD_50_ (301.69 ± 11.81 ms vs. 262.18 ± 9.74 ms, *p* < 0.05; 301.69 ± 11.81 ms vs. 256.98 ± 9.70 ms, *p* < 0.01) and APD_90_ (430.73 ± 16.64 ms vs. 385.82 ± 10.82 ms, *p* < 0.05; 430.73 ± 16.64 ms vs. 378.57 ± 12.01 ms, *p* < 0.01), while exerting no significant effect on APD_30_ (Figure 3D–F). The above-mentioned effect was reversed after the drug was washed out.

**Figure 4:**
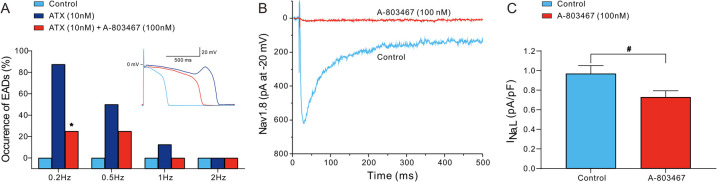
A-803467 inhibits early afterdepolarizations (EADs) and Nav1.8 induced I_Na,L_ in Purkinje fibers and cells (*N* = number of dogs; *n* = number of tissue slices or cells). **(A)** The incidence of EADs in Purkinje fibers exposed to ATX-II (10 nmol/L) alone or in combination with 100 nmol/L A-803467. The inset shows an example of A-803467 suppressing the EAD elicited by exposure to ATX-II at 0.5 Hz. *p < 0.05 vs. control + ATX (*N* = 8 and *n* = 8, respectively). **(B)** 100 nmol/L A-803467 inhibits *SCN10A*-mediated I_Na,L_ in transfected ND7/23 cells. **(C)** Summary data of relative I_Na,L_ at −20 mV in the absence (control) and presence of 100 nmol/L A-803467 in Purkinje cells. ^#^*p* < 0.05 vs. control (*N* = 6 and *n* = 8, respectively).

The excessive prolongation of APs caused by the enhancement of I_Na,L_ can lead to EADs, which are associated with arrhythmogenesis in both congenital and acquired disease conditions, such as LQTS, heart failure, myocardial hypertrophy and ischemia-reperfusion. We examined the effects of 100 nmol/L A-803467 on EADs in Purkinje fibers triggered by the I_Na,L_ agonist ATX-II at a concentration of 10 nmol/L. As depicted in the inset of Figure 4A, ATX-II significantly prolonged APD and elicited EAD at a BCL of 2,000 ms. The administration of A-803467 attenuated APD prolongation induced by ATX-II and successfully suppressed the occurrence of EAD. Figure 4A summarizes the development of EADs in 8 Purkinje fibers exposed to 10 nmol/L ATX-II alone or together with 100 nmol/L A-803467 at different stimulation rates. At stimulation rates of 1 and 2 Hz, ATX-II barely failed to induce EADs. At a slower stimulation rate of 0.5 Hz, EADs developed in 2 of 8 (25%) Purkinje fibers following the addition of A-803467. In the absence of A-803467, EADs were induced by ATX-II in 4 of 8 (50%) Purkinje preparations. However, this reduction did not achieve the statistical significance. The incidence of EADs elicited by ATX-II was significantly reduced by A-803467 at a slower stimulation rate of 0.2 Hz (7/8 vs. 2/8, *p* < 0.05). To confirm that the underlying antiarrhythmic effects of A-803467 were mediated by inhibition of I_Na,L_, we first examined its effects on *SCN10A*-encoded Nav1.8 channels heterologously expressed in ND7/23 cells. A-803467 (100 nmol/L) significantly inhibited I_Na,L_ in ND7/23 cells (Figure 4B). Furthermore, A-803467 also significantly reduced I_Na,L_ in Purkinje cells from 0.97 ± 0.08 pA/pF to 0.73 ± 0.06 pA/pF at 0.2 Hz (*p* < 0.01, Figure 4C).

## Discussion

*SCN10A* is located on human chromosome 3p22, adjacent to *SCN5A*. Nav1.8 shares 70.4% protein sequence similarity with Nav1.5 and plays a crucial role in driving the upstroke of APs and facilitating conduction in peripheral nerves (18). The role of *SCN10A* in the heart began to attract attention following initial genome-wide association studies that revealed its association with cardiac conduction. *SCN10A*, like its high expression in peripheral neurons, is also readily detectable in intracardiac neurons, primarily cholinergic neurons (16). Chambers et al. detected *SCN10A* mRNA expression in both the atria and ventricles of mouse cardiac tissue, a finding further corroborated in isolated atrial and ventricular myocytes from wild-type mice (11). Subsequent studies documented *SCN10A* mRNA expression in ventricular myocytes and Purkinje fiber of mice, humans, and rabbits (4, 5, 12–15, 19–22). However, some studies also exhibited divergent results, failing to detect mRNA or full-length mRNA in ventricles or ventricular myocytes (7, 23, 24). This discrepancy may be attributed to variations in experimental conditions, expression levels below detection thresholds, high rates of alternative splicing, or methodological differences (primer position in quantitative polymerase chain reaction etc.) (14, 19, 23, 25). More recently, Man et al. reported that the full-length *SCN10A* transcript was undetectable in the ventricles of both mouse and human heart using quantitative reverse transcription polymerase chain reaction, although short *SCN10A* transcripts containing the final seven exons was detected (25). Similar findings were reported via RNA sequencing (7). By designing primers for individual exons to address alternative splicing, Dybkova et al. successfully detected *SCN10A* mRNA expression in both human left ventricular myocardium and isolated failing cardiomyocytes (22). Nav1.8 protein expression has been observed in ventricular myocardium or cardiomyocytes from mice, rats, humans, and canines via immunohistochemistry and/or Western blot techniques (12–14, 22, 26–28). In line with *SCN10A* mRNA and protein expression, Nav1.8-mediated I_Na,L_ in ventricular myocytes is well recognized under both physiological conditions and pharmacological provocation (13, 15, 22, 23, 29, 30). Moreover, polymorphic variants of *SCN10A* have been linked to the QT interval, with rare variants identified in patients with LQTS (6, 31). Collectively, these findings substantiate the presence of Nav1.8 in ventricular myocardium, although at significantly lower expression levels than Nav1.5, both at the mRNA and protein levels.

Our findings demonstrated that A-803467 did not exert significant effects on the APA, Vmax, and MDP in epicardial cells, M cells, or Purkinje cells. Considering that Vmax and APA serve as indicators of rapid Na^+^ influx and peak Na^+^ current, our experimental data suggest that Na_V_1.8 makes a negligible contribution to the peak Na^+^ current in ventricular cardiomyocytes. This observation concurs with prior studies showing that neither specific pharmacological inhibition nor genetic ablation of Nav1.8 affects peak Na^+^ current or its kinetics in either atrial or ventricular cardiomyocytes (13, 15, 23, 30). Our data revealed that non-cardiac sodium channels contribute approximately 10% of the peak Na^+^ current in canine cardiomyocytes, implying that Nav1.8's contribution to the overall peak sodium current is likely minimal (32). Peak Na^+^ current is a major determinant of cardiac conduction velocity. The lack of significant effect on Vmax upon Nav1.8 inhibition suggests that Nav1.8 may not directly influence cardiac conduction through its electrophysiological effects in cardiomyocytes. However, it is noteworthy that Macri et al. identified a negative correlation between Nav1.8-dependent I_Na,L_ and PR intervals in a large-scale genetic screen (5). Additionally, Nav1.8 may modulate cardiac conduction indirectly by modulating Nav1.5 or via effects on intracardiac neurons (33).

I_Na,L_ persists long after peak Na^+^ current is inactivated, substantially influencing the shape and duration of the AP (34). Our group previously demonstrated that non-cardiac Nav isoforms contribute approximately 50% of I_Na,L_ in canine ventricular cardiomyocytes under physiological conditions (32). Nav1.8 manifests a more depolarized voltage dependence of inactivation and slower inactivation kinetics compared to Nav1.5 (7). Furthermore, I_Na,L_ mediated by Nav1.8 is considerably greater than that mediated by Nav1.5 in heterologous system (15, 35). These outcomes align with numerous studies indicating that Nav1.8 mainly contributes to the I_Na,L_ (5, 13, 15, 22, 23, 29, 36). We observed that A-803467 had no effect on APD in epicardial cells. In contrast, it significantly reduced APD_50_ and APD_90_ in M cells and Purkinje fiber. This discrepancy suggests that the distribution of Nav1.8 and the associated I_Na,L_ is heterogeneous across different cardiac cell types. Indeed, canine M cells and Purkinje fibers exhibit a larger I_Na,L_ amplitude compared to epicardial cells, and this more prominent I_Na,L_ contributes to their longer APD and steeper rate dependence of APD (34, 37, 38) Previous studies have demonstrated enrichment of *SCN10A* mRNA in the cardiac conduction system and Purkinje fibers during both developmental stages and in the adult murine heart (4, 20, 25). Vassalle et al. discovered a slowly inactivating sodium current in canine Purkinje cells that contributes to APD prolongation, while being minimally present or absent in ventricular cardiomyocytes. Remarkably, about 30% of the slowly inactivating sodium current remained even in the presence of 30 µmol/L tetrodotoxin, indicating that Nav1.8 might be responsible for this current (39). Yang et al. discovered that Nav1.8-mediated I_Na,L_ varies among different ventricular cardiomyocytes, leading to inconsistent APD shortening effects of A-803467, which was observed in only 50% of cells (15). Casini et al. observed that treatment with A-803467 induced a slight but statistically significant reduction in both APD_50_ and APD_90_ in rabbit ventricular cardiomyocytes, although this effect was only partially reversible in a minority of cells after washout. Of note, A-803467 treatment significantly reduced APD_50_ in human-induced pluripotent stem cell-derived cardiomyocytes, with the effect being reversible upon washout in the majority of cells, although the blocker did not alter APD in every cell (19). Verkert et al. reported that the expression of Nav1.8 was also heterogeneous in intracardiac neurons (16). Similarly, other ionic channels such as I_ks_ and I_to_ are also not uniformly distributed across the ventricular wall (40, 41).

Some researchers were unable to detect functional Nav1.8-mediated I_Na,L_ (16, 19). The discrepancy potentially arises from cell-specific variations in Nav1.8 expression. As we have described, Nav1.8 expression levels differ among cell types, and the low expression in certain cells might preclude the detection of Nav1.8-mediated I_Na,L_. Additionally, this discrepancy could also stem from variations in experimental conditions, such as temperature differences, the use of perforated patch vs. ruptured patch techniques, and variations in recording solutions. A previous study demonstrated that I_Na,L_ was markedly elevated in human right atrial cardiomyocytes from AF patients compared to those from patients in sinus rhythm at room temperature. However, this disparity in I_Na,L_ amplitudes between AF and sinus rhythm cells diminished and statistically insignificant at physiological temperature (42). Similarly, I_Na,L_ in human-induced pluripotent stem cell-derived cardiomyocytes decreased with increasing temperature (43). We found that A-803467 shortened APD in M cells and Purkinje cells only at slow pacing rates, which aligns with previous studies. Notably, Yang et al. and Bengel et al. reported similar rate-dependent APD shortening by A-803467 in mouse ventricular myocytes, with the latter specifically demonstrating this effect in ATX-II stimulated preparations. Importantly, this phenomenon was abolished in *SCN10A* knockout models (15, 29, 33). Li et al. reported that I_Na,L_ was frequency-dependent, with its amplitude increasing as the pacing frequency decreased. Notably, Purkinje cells displayed a significantly larger rate dependence of I_Na,L_ compared to ventricular myocytes (44). This characteristic may explain why A-803467 shortens APD only under slow pacing rates.

The distinctive properties of Purkinje fibers-including their complex anatomical structure, excitation-contraction coupling modes, and unique electrophysiological characteristics such as AP morphology, APD, and the intricate interplay of membrane currents render them particularly susceptible to afterdepolarizations and ventricular arrhythmias (40, 45). Accumulating evidence has demonstrated that ablation of Purkinje fiber-originated triggers effectively prevents or reduces the recurrence of ventricular tachycardia/fibrillation across a spectrum of cardiac conditions, encompassing both inherited disorders (Brugada syndrome, early repolarization syndrome, LQTS and idiopathic ventricular fibrillation) as well as acquired conditions such as ischemic heart disease (46, 47). Enhanced I_Na,L_ underlies the generation of the afterdepolarizations and triggered activity in Purkinje cells (44, 48). ATX-II can enhances the I_Na,L_ generated by multiple voltage-gated sodium channels, including Nav1.8, resulting in APD prolongation that can trigger EADs. We reported that in Purkinje fibers, A-803467 showed a tendency to inhibit ATX-II induced EADs at a pacing frequency of 0.5 Hz, with significant suppression of EADs observed at a lower frequency of 0.2 Hz via reduction of I_Na,L_. These findings provide compelling evidence that Nav1.8-mediated I_Na,L_ represents a promising therapeutic target for the pharmacological management of bradycardia associated arrhythmias. Yang et al. illustrated A-803467 effectively eliminated ATX-II induced EADs and trigged activity in mouse and rabbit ventricular cardiomyocytes (15). Furthermore, an enhanced I_Na,L_ can precipitate intracellular Ca^2+^ overload by activating the reverse mode of the Na^+^/Ca^2+^ exchanger. This may lead to diastolic sarcoplasmic reticulum Ca^2+^ leak (Ca^2+^ sparks) and delayed afterdepolarizations due to increased Ca^2+^/calmodulin-dependent protein kinase II (CaMKII)-dependent ryanodine receptor 2 phosphorylation. Ca^2+^ sparks are considered as a crucial substrate for delayed afterdepolarizations and triggered arrhythmias (13, 48). In mouse ventricular cardiomyocytes stimulated by ATX-II, pharmacological inhibition of Nav1.8 significantly reduced I_Na,L_, thereby diminishing reverse mode Na^+^/Ca^2+^ exchanger current, diastolic sarcoplasmic reticulum Ca^2+^ leak and CaMKII-dependent phosphorylation of ryanodine receptor 2 (29). In human ventricular cardiomyocytes from hearts with failure and hypertrophy, both mRNA and protein expression levels of Nav1.8, as well as the I_Na,L_ mediated by Nav1.8, were found to be significantly increased. Inhibition of Nav1.8 notably reduced I_Na,L_ and diastolic sarcoplasmic reticulum Ca^2+^ leak (22, 49). Moreover, in a chronic CaMKIIδc overexpression heart failure mouse model, genetic ablation of *SCN10A* significantly suppressed I_Na,L_, reduced Ca^2+^-dependent proarrhythmic triggers and delayed afterdepolarizations in ventricular myocytes, and decreased *in vivo* arrhythmia events (13). Wu et al. revealed endogenous I_Na,L_ underlies the reverse rate-dependent prolongation of APD induced by IKr inhibitors, enhances beat-to-beat repolarization variability, and promotes bradycardia-associated ventricular arrhythmias (50). Our findings further illustrated that A-803467 significantly reduced APD and suppressed EADs through I_Na,L_ inhibition under slow pacing conditions. During bradycardia, particularly in pathological conditions, such as myocardial infarction, heart failure, and ventricular hypertrophy, the pathological upregulation of Nav1.8-mediated I_Na,L_ significantly elevates the risk of arrhythmias at slow heart rates, with this effect being most prominent in M cells and Purkinje fibers. This electrophysiological paradigm elucidates Nav1.8 channels as a compelling therapeutic target in the pharmacological management of bradycardia-associated arrhythmogenesis.

The underlying mechanisms by which *SCN10A* modulates cardiac electrophysiology in ventricular myocytes are not yet fully elucidated. Beyond the direct electrophysiological effects of Nav1.8 on ventricular cardiomyocyte, van den Boogaard et al. proposed that intronic variants in *SCN10A* altered the transcription factor binding site for TBX5 and TBX3, thereby regulating the expression of *SCN5A* (51). Additionally, our group previously demonstrated that in a heterologous expression system, the co-expression of *SCN10A* with *SCN3B* yielded a negligible sodium current. In contrast, co-expressing *SCN10A* with both *SCN5A* and *SCN3B* nearly doubled the mediated sodium current compared to co-expression of *SCN5A* and *SCN3B*. Co-immunoprecipitation assays further revealed an association between Nav1.5 and Nav1.8 when co-expressed (3, 52). Moreover, when short *SCN10A* was co-expressed with *SCN5A* in heterologous expression system, nearly identical results were observed (25). These findings support the premise that Nav1.8 may affect the electrophysiology of cardiomyocytes either through direct physical interactions or indirect interactions with Nav1.5 as a protein complex. Additional evidence further supports this hypothesis. *in situ* hybridization analysis revealed that *SCN10A* and *SCN5A* exhibited overlapping spatial and temporal expression patterns within the mouse heart (21). Besides, CaMKIIδc co-immunoprecipitated with Nav1.8 in human ventricular myocardium from both non-failing and heart failure samples. Immunofluorescence staining further corroborated co-localization of CaMKIIδc and Nav1.8 in isolated human ventricular cardiomyocytes (13). Moreover, it is also well established that CaMKIIδc interacts with Nav1.5 as part of a macro complex. However, evidence for such a protein complex *in vivo* is currently lacking, and further studies are needed to confirm this hypothesis. What's more, *SCN10A* can also indirectly modulate the electrophysiology of cardiomyocytes through intracardiac neurons (33).

### Limitations

The experimental data were derived from canine tissue, and the cell type-specific results obtained from our particular canine model may limit the generalizability of our findings to other species or humans. Therefore, clinical extrapolation should be approached with caution. Further validation in more physiologically relevant models, including arterially perfused ventricular wedge preparations and *in vivo* studies is warranted. Additionally, given the known heterogeneous distribution of *SCN10A* between ventricles, the applicability of our results, obtained from left ventricular tissue, to the right ventricle and whole heart requires further exploration. Although A-803467 is currently the most selective Nav1.8 blocker available, the possibility of off-target effects on other sodium channels cannot be entirely ruled out.

## Conclusion

Nav1.8 exhibits a heterogeneous role in cardiac electrophysiology across the ventricular wall. Nav1.8 inhibition in Purkinje fibers attenuates I_Na,L_, shortens APD, and suppresses EADs, highlighting its potential as a therapeutic target for arrhythmias associated with bradycardia.

## Data Availability

The original contributions presented in the study are included in the article/Supplementary Material, further inquiries can be directed to the corresponding authors.
